# Lifetime suicidal thoughts, attempts, and lethality of attempts as major outcome domains of psychotic disorders: a 21-year prospective cohort study after a first-episode psychosis – CORRIGENDUM

**DOI:** 10.1017/S0033291725001059

**Published:** 2025-05-05

**Authors:** Victor Peralta, Lucía Moreno-Izco, Elena García de Jalón, Ana M. Sánchez-Torres, David Peralta, Lucía Janda, Manuel J. Cuesta, SEGPEPs Group

**Affiliations:** 1 Mental Health Department, Servicio Navarro de Salud, Pamplona, Spain; 2 Instituto de Investigación Sanitaria de Navarra (IdiSNA), Pamplona, Spain; 3 Department of Psychiatry, Hospital Universitario de Navarra, Pamplona, Spain; 4 Departamento de Ciencias de la Salud, Universidad Pública de Navarra (UPNA), Pamplona, España

When this article published in Psychological Medicine it contained an error in the Abstract. In the Abstract it is stated that “Lethality was independently predicted by familial load of major depression, obstetric complications, neurodevelopmental delay, and poor adolescence social networks”. Instead of obstetric complications it should have been male sex. Thus, the correct statement is as follows: “Lethality was independently predicted by familial load of major depression, male sex, neurodevelopmental delay, and poor adolescence social networks”.

This article was also published listing the members of the authorship group SEGPEPs in the main author list. This has now been updated so that they’re listed in the Acknowledgements section.

The funding statement for this article has also been updated to the following:

“This work was funded by the Instituto de Salud Carlos III, the Spanish Ministry of Science, Innovation and Universities, the European Regional Development Fund (ERDF/FEDER) (VP, gran numbers PI 16/02148; EGJ and MJC, PI 19/01968), and the Regional Government of Navarra (VP, grant number 31/17; LMI, grant number 41/18).”

The authors apologise for these errors.
